# Impact of Surgical Timing on Outcome in Papillary Thyroid Cancer Patients in Saudi Arabia

**DOI:** 10.3390/jcm14248662

**Published:** 2025-12-06

**Authors:** Omar Zaki Alaidaroos, Saleh F. Aldhahri, Talal Banan Alanazi, Malak Satam Alanazi, Khalid H. Alqahtani, Mohammed Alessa, Naif Fnais, Faisal R. Alzahrani

**Affiliations:** 1 Department of Otorhinolaryngology-Head and Neck Surgery, College of Medicine, King Saud University, Riyadh 11451, Saudi Arabiaaddhahri@gmail.com (S.F.A.); kalqahtani@ksu.edu.sa (K.H.A.); malessa@ksu.edu.sa (M.A.); nfnais@ksu.edu.sa (N.F.); fralzahrani@ksu.edu.sa (F.R.A.); 2 Department of Otorhinolaryngology-Head and Neck Surgery, King Fahad Medical City, Riyadh 11525, Saudi Arabia; tbsatb@gmail.com; 3 Department of Otolaryngology-Head and Neck Surgery, King Fahad University Hospital, AL Khobar 31952, Saudi Arabia; 4 King Abdullah International Medical Research Center, National Guard Health Affairs, King Abdulaziz Medical City, Riyadh 11426, Saudi Arabia

**Keywords:** carcinoma, papillary, differentiated, histopathology, thyroid, neoplasms, surgery, thyroidectomy

## Abstract

**Background/Objectives:** To evaluate whether the interval between diagnosis and surgery is associated with aggressive histopathological features in papillary thyroid carcinoma (PTC) among patients treated at a tertiary hospitals in Saudi Arabia. **Methods:** This retrospective study was conducted at King Fahad Medical City, Riyadh, Saudi Arabia, from November 2009 to November 2024. A total of 350 adult patients who underwent surgical intervention for PTC were included based on predefined inclusion and exclusion criteria. Data were collected from medical records and included demographic, clinical, and histopathological characteristics. The primary outcome was the presence of aggressive histopathological features, assessed through tumor subtype, tumor size, extrathyroidal extension (ETE), lymph-node involvement, extracapsular extension (ECE), multifocality, goitrous tumor, thyroiditis, perineural invasion, lymphovascular invasion, and margin status. Surgical timing was categorized into two groups: less than six months and six months or more from diagnosis. **Results:** Most patients were female (76.6%), with a mean age of 40.7 years, and 78.3% were classified as overweight or obese. The analysis showed no statistically significant differences in histopathological outcomes between the two surgical timing groups. A significant association was found between positive lymph-node involvement and surgery performed within the first six months of diagnosis (*p* = 0.004). **Conclusions:** This study showed that surgical timing does not significantly affect histopathological outcomes in papillary thyroid carcinoma, particularly in patients without aggressive disease features. The association between early surgery and positive lymph-node involvement may reflect the prioritization of patients with preoperative lymph-node disease for earlier surgical intervention, highlighting the importance of individualized treatment planning. Further research is needed to explore the long-term impact of surgical timing and potential subgroup differences.

## 1. Introduction

Thyroid cancer is the most common endocrine malignancy, accounting for approximately 3.5% of all cancers worldwide [1]. Papillary thyroid carcinoma (PTC) is the predominant histologic subtype, followed by follicular, medullary, and anaplastic carcinomas [2]. In Saudi Arabia, 23,846 thyroid cancer cases were diagnosed between 1990 and 2019, and it is currently the third most common cancer, with an estimated mortality of 1.5% [3,4]. Despite the generally favorable prognosis of PTC, evidence regarding the impact of surgical delay remains limited. During the COVID-19 pandemic, several reports suggested that many cases of differentiated thyroid cancer could be safely deferred in the absence of airway compromise [5]. Additionally, some studies have reported that delaying surgery in patients with papillary thyroid microcarcinoma (PTMC) is not associated with a higher risk of structural recurrence or persistent disease compared with immediate surgery [6].

In addition, some studies suggested that delayed surgeries in papillary thyroid microcarcinoma (PTMC) patients was not associated with a higher risk of structural, recurrence or persistent disease compared to immediate surgery [7], and the timing of surgery does not seem to affect short-term disease outcomes in intermediate- to high-risk PTC patients [8]. However, there are other studies that indicate delays in surgery for PTC patients may impact overall survival (OS) and disease-specific survival (DSS), particularly in localized disease before nodal metastasis occurs [9,10]. The prognosis of papillary thyroid cancer patients can be predicted using the “American Thyroid Association (ATA) risk stratification system” and the “AJCC/TNM system”. The ATA system is designed to predict disease recurrence, while the AJCC/TNM system is optimized to predict survival in patients with thyroid cancer. Both systems depend heavily on histopathological findings [11,12].

The objective of this study is to examine the relationship between surgical timing and histopathological findings in patients with papillary thyroid carcinoma. The optimal timing for surgical intervention in PTC remains insufficiently studied. We hypothesize that a surgical delay beyond six months from diagnosis is associated with more adverse histopathological characteristics.

## 2. Methods

### 2.1. Study Design and Setting

A retrospective cohort study was conducted at a tertiary hospital in Riyadh, Saudi Arabia, from November 2009 to November 2024.

### 2.2. Study Population

Patients who underwent thyroid surgery at King Fahad Medical City, a large tertiary hospital in the central region of Saudi Arabia, and had a confirmed diagnosis of papillary thyroid carcinoma (PTC) based on final histopathology were identified from the hospital database.

Inclusion Criteria:

- Adult patients (≥18 years) with PTC.
- Patients who underwent thyroid surgery as primary treatment.
- Availability of preoperative FNA results and final histopathological evaluation.

Exclusion Criteria:

1. Non-papillary thyroid cancers or other thyroid disorders.
2. Papillary thyroid microcarcinoma (≤1 cm), due to its typically indolent behavior and low risk of aggressive histopathological features.

After applying the inclusion and exclusion criteria, 350 patients were included in the final analysis.

### 2.3. Data Collection

Data were collected retrospectively from electronic medical records and hospital databases. The variables were organized into five categories—demographic, clinical, laboratory, surgical, and histopathological—and entered into an Excel sheet before analysis. All entries were reviewed for accuracy.

Patients who underwent multiple thyroid surgeries were identified, and only data from the initial surgery, in which PTC was diagnosed, were retained because they provided the most accurate information about the primary tumor. Data from subsequent surgeries were excluded to ensure the analysis focused on the main tumor and its pathological characteristics.

Patients with incomplete or missing data relevant to the study variables were removed using complete-case analysis; missing values were not imputed. Duplicate entries were also excluded.

### 2.4. Study Variables and Outcome Measures

The primary outcome was the presence of aggressive histopathological features, assessed using the following characteristics: tumor type and subtype, tumor size, extrathyroidal extension (ETE), positive lymph nodes, extracapsular extension (ECE), multifocality, goitrous tumor, thyroiditis, perineural invasion, lymphovascular invasion, and margin status.

The primary predictor was time to surgery, defined as the interval (in days) between diagnosis by FNA and the date of surgery. Time to surgery was categorized into two groups: <6 months and ≥6 months.

A 6-month cutoff was selected based on a large cohort study of 8170 PTC patients (Chaves et al., 2023 [10]), which reported worse overall and disease-specific survival when surgery was delayed beyond six months. This threshold was used to evaluate whether prolonged time to surgery affects histopathological features.

Additional variables included:

▪ Age (years)
▪ Sex (male/female)
▪ Body mass index (BMI, kg/m^2^): calculated as weight (kg) divided by height squared (m^2^). BMI was categorized according to World Health Organization guidelines:
  - Underweight: <18.5
  - Normal weight: 18.5–24.9
  - Overweight: 25.0–29.9
  - Obesity class I: 30.0–34.9
  - Obesity class II: 35.0–39.9
  - Obesity class III: ≥40.0
▪ Thyroid function: categorized based on preoperative laboratory results:
  - Euthyroid: normal TSH and free T4
  - Hypothyroid: elevated TSH and/or low free T4
  - Hyperthyroid: suppressed TSH and/or elevated free T4
▪ FNA dates and results
▪ Time and type of surgery

### 2.5. Data Analysis

Statistical analysis was conducted using SPSS version 29 Descriptive statistics were reported as frequencies for categorical variables and as mean ± standard deviation (SD) for continuous variables such as age. Shapiro–Wilk testing was used to assess normality.

Chi-square tests evaluated differences in categorical variables, and multivariate logistic regression was used to examine predictors of selected aggressive features (lymph node positivity and ETE). A *p*-value <0.05 was considered statistically significant.

### 2.6. Ethical Considerations

The study was approved by the Institutional Review Board of King Fahad Medical City. Informed consent was waived because of the retrospective design and the use of de-identified data. All collected data were handled anonymously and treated confidentially, and were used solely for research purposes.

## 3. Results

“A total of 350 papillary thyroid carcinoma cases met the inclusion criteria and were analyzed.”

Table 1 summarizes the demographic and clinical characteristics of the included patients. Most patients were female (76.6%), and 86.9% were younger than 55 years, with a mean age of 40.7 ± 13.4 years. Regarding BMI, 78.3% were classified as overweight (30.9%), obese (26.9%), or morbidly obese (20.5%), while only 21.7% had a normal BMI. Most patients were euthyroid (83.4%), whereas 6.3% were hypothyroid and 10.3% were hyperthyroid.

With respect to time to surgery, 74.9% underwent surgery within six months of diagnosis by FNA, whereas 25.1% had a delay of six months or more. Total thyroidectomy (TT) was the most common procedure (60.3%), followed by TT with central neck dissection (CND, 9.4%) and TT with unilateral neck dissection (UND, 10.3%). Hemithyroidectomy was rare (right: 2.3%; left: 2.3%).

Table 2 presents the results of the initial FNA. Papillary thyroid carcinoma (PTC) was the most common diagnosis (37.1%), followed by cases categorized as “suspicious for PTC” (18.0%) and “atypia of undetermined significance (AUS)” (17.4%). Benign nodules represented 16.6% of findings, and 8.0% were reported as follicular neoplasms. Non-diagnostic samples accounted for 2.9%, which falls within the acceptable range reported in the literature.

Table 3 summarizes the histopathological findings. Most tumors were classified as classical PTC (55.1%), followed by the follicular variant (27.4%). Mixed subtypes (e.g., classical and follicular variant) accounted for 8.9%, while uncommon variants such as oncocytic or tall cell types represented less than 3%. Thyroiditis was present in 46.6% of cases, and goiter was identified in 39.7%.

Approximately one-third of cases demonstrated extrathyroidal extension (ETE, 33.1%) and extracapsular extension (ECE, 36.9%), both considered adverse prognostic indicators. Lymph node involvement was reported in 34.3% of patients, and lymphovascular invasion in 20.0%. Neural invasion was rare (0.9%) but reflects aggressive tumor behavior.

Negative surgical margins were achieved in 50.0% of cases, whereas 29.9% had close margins and 20.1% had positive margins. Multifocality was observed in 50.1% of cases, while 49.9% were unifocal. Tumor size was <2 cm in 42.6% of patients, 2–<4 cm in 37.4%, and ≥4 cm in 20.0%, indicating early-stage disease in most patients but advanced disease in a significant minority.

Table 4 evaluates the relationship between time from diagnosis to surgery and clinical characteristics. No statistically significant difference in surgical timing was observed based on the presence of thyroiditis. Although goiter-positive cases more frequently underwent surgery after six months (30.2%), this difference was not statistically significant.

A significant proportion of patients with lymph node (LN) involvement underwent surgery within six months (84.2%) compared with those who experienced delays ≥6 months (15.8%) (*p* = 0.004; OR = 1.88, 95% CI: 1.19–2.97), suggesting a trend toward expedited surgical intervention in more advanced disease. No statistically significant differences were found in extracapsular extension (ECE) or lymphovascular invasion with respect to time to surgery.

A strong statistical association was observed between initial FNA results and time to surgery (*p* < 0.001). Patients diagnosed with PTC (91.5%) or follicular neoplasms (92.9%) predominantly underwent surgery within six months. In contrast, patients with benign findings (34.5%) or atypia of undetermined significance (AUS) (59.0%) were more likely to experience delays, reflecting a clinical tendency to monitor indeterminate or benign nodules while prioritizing confirmed or suspected malignancies. Tumor size distribution did not differ significantly between the timing groups. Similarly, margin status showed no statistically significant association with time to surgery (*p* = 0.589).

Table 5 presents the logistic regression model evaluating predictors of lymph node (LN) involvement in PTC. FNA cytology emerged as the strongest predictor. Compared with PTC, the benign (OR = 0.711, 95% CI: 0.620–0.816, *p* < 0.001), AUS (OR = 0.701, 95% CI: 0.611–0.803, *p* < 0.001), and follicular neoplasm (OR = 0.699, 95% CI: 0.583–0.838, *p* < 0.001) categories were each associated with nearly a 30% lower likelihood of LN involvement.

Patients aged ≥55 years demonstrated a higher likelihood of LN involvement—approximately 15% greater—compared with younger patients (OR = 1.150, 95% CI: 0.999–1.324), with borderline statistical significance (*p* = 0.052). Although larger tumor size and higher BMI showed trends toward increased LN involvement, these associations were not statistically significant.

Table 6 shows that patients aged ≥55 years had 27.3% higher odds of extrathyroidal extension (ETE) compared with those <55 years (OR = 1.273, 95% CI: 1.106–1.465, *p* < 0.001). Tumor size was also a strong predictor of ETE. Relative to tumors <2 cm, tumors ≥4 cm were associated with a 28.6% increase in the odds of ETE (OR = 1.286, 95% CI: 1.133–1.459, *p* < 0.001), while tumors 2–4 cm showed a 23.0% increase (OR = 1.230, 95% CI: 1.108–1.366, *p* < 0.001).

Compared with PTC, benign cytology was associated with a 26.5% reduction in the odds of ETE (OR = 0.735, 95% CI: 0.640–0.843, *p* < 0.001), AUS with a 23.9% reduction (OR = 0.761, 95% CI: 0.664–0.871, *p* < 0.001), and follicular neoplasm with a 26.1% reduction (OR = 0.739, 95% CI: 0.617–0.885, *p* = 0.001). Cases categorized as “suspicious for PTC” showed a smaller but still significant reduction of 13.8% (OR = 0.862, 95% CI: 0.754–0.985, *p* = 0.030). Non-diagnostic cytology showed no statistically significant association with ETE.

## 4. Discussion

In this cohort of 350 patients with papillary thyroid carcinoma (PTC), the demographic profile characterized by a predominance of female (76.6%) age distribution in this study are consistent with findings from previous research, reflecting the well-documented gender and age-related patterns in papillary thyroid carcinoma (PTC) incidence [13,14,15].

The high prevalence of elevated Body Mass Index (BMI) (78.3%) also aligns with previous literature; however, The prevalence of elevated Body Mass Index (BMI) (78.3%) among patients with PTC in our cohort aligns with existing literature that highlights the association between increased BMI and thyroid cancer.

Elevated BMI has also been associated with more aggressive tumor behavior and worse outcomes in PTC patients [16]. Concerning our primary objective of assessing whether surgical timing affects the histopathological outcomes of PTC patients and, consequently their survival and recurrence we found no statistically significant difference in adverse histopathological features between the two surgical timing groups. This finding is consistent with Zhou et al., who concluded that the timing of surgery does not significantly influence short-term disease outcomes in intermediate- to high-risk PTC patients [8].

However, our results diverge from some studies that suggest delayed surgical intervention may adversely affect overall survival and disease-specific survival [9,10]. Interestingly, there was a significant association (*p* = 0.004) between positive lymph node involvement on histopathology and undergoing surgery within the first six months. This finding may reflect the prioritization of patients with preoperative lymph node involvement or more advanced disease for earlier surgical intervention, highlighting the importance of individualized treatment planning. The lack of significant impact of surgical timing on adverse histopathological features in this study found that, in the absence of overtly aggressive disease features, delays in surgery might not compromise patient outcomes. This could provide reassurance in settings with long surgical waiting lists or resource constraints. However, these findings should be interpreted cautiously. It is possible that specific subgroups, such as those with more advanced disease may experience different outcomes with delayed intervention.

### 4.1. Limitations

This study has several limitations. First, its retrospective design relies on medical records, and we did not assess non-medical reasons for surgical delay, such as patient preference, socioeconomic factors, logistical constraints, or institutional scheduling issues. These unmeasured factors may have influenced both the timing of surgery and the pathological findings. Second, the study lacks long-term follow-up data, including recurrence and survival outcomes, and therefore cannot evaluate the prognostic implications of surgical timing. Finaly, selection bias is likely, as patients with clinically suspicious or advanced disease were more frequently prioritized for earlier surgery, which may have impacted group comparisons.

### 4.2. Future Directions

Future research should aim to validate these findings in larger, multi-institutional cohorts with extended follow-up to better evaluate the long-term impact of surgical timing on recurrence and survival. Prospective studies focusing on specific patient subgroups are particularly needed to determine whether certain populations are more vulnerable to delays in surgical intervention. Additionally, further investigation into the role of preoperative risk-stratification systems in guiding the urgency of surgery could support more tailored and evidence-based treatment decision-making.

## 5. Conclusions

This study showed that surgical timing does not significantly influence histopathological outcomes in papillary thyroid carcinoma, particularly among patients without aggressive disease features. The observed association between earlier surgery and positive lymph node involvement likely reflects the prioritization of patients with suspected or confirmed nodal disease for prompt intervention, underscoring the importance of individualized treatment planning. Further research is warranted to examine the long-term effects of surgical timing and to determine whether specific patient subgroups may be differentially affected.

## Figures and Tables

**Table 1:** Demographic and clinical characteristics of the cases (n = 350).

Characteristics	N	%
Gender		
Male	82	23.4%
Female	268	76.6%
Age categories		
<55 years	304	86.9%
≥55 years	46	13.1%
Mean ± SD	40.7 ± 13.4	
BMI categories		
Within normal	76	21.7%
Overweight	108	30.9%
Obese	94	26.9%
Morbid obesity	72	20.5%
Thyroid function		
Euthyroid	292	83.4%
Hypothyroid	22	6.3%
Hyperthyroid	36	10.3%
Duration between diagnosis and surgery		
<6 months	262	74.9%
≥6 months	88	25.1%
Type of surgery		
TT	211	60.3%
TT and CND	33	9.4%
TT and UND	36	10.3%
TT and BND	17	4.9%
Comp	36	10.3%
Comp and CND	2	0.6%
Comp and UND	1	0.3%
Comp and BND	6	1.7%
Right hemithyroidectomy	8	2.3%
Left hemithyroidectomy	8	2.3%

TT (Total Thyroidectomy), CND (Central Neck Dissection), UND (Unilateral Neck Dissection), BND (Bilateral Neck Dissection), Comp (Completion Thyroidectomy).

**Table 2:** Results of the FNA.

FNA Results	N	%
PTC	130	37.1%
Suspecouic for PTC	63	18.0%
Atypia of Undetermined Significance (AUS)	61	17.4%
Benign	58	16.6%
Follicular Neoplasm	28	8.0%
Not diagnostic	10	2.9%

PTC (Papillary Thyroid Carcinoma).

**Table 3:** Histopathological results of the cases (n = 350).

Histopathology of the Tumor	N	%
Pathology type		
PTC Classical	193	55.1%
PTC follicular variant	96	27.4%
PTC Classical and PTC follicular variant	33	8.9
PTC Classical and PTC oncocytic variant	9	2.7
PTC Classical and other PTC variants	8	2.3
PTC oncocytic variant	5	1.4
Other PTC variants (Insular, Tall cell, Columnar, diffuse sclerosing … etc.)	4	1.1
PTC follicular variants and other PTC variants	4	1.1
Tumor related features		
Thyroiditis	161	46.6%
Goitrous	139	39.7%
ETE (Extrathyroidal Extension)	116	33.1%
ECE (Extracapsular Extension)	129	36.9%
LN (Lymph Node Involvement)	120	34.3%
Neural invasion	3	0.9%
Lymphovascular invasion	70	20.0%
Margin (n = 334)		
Negative	167	50.0%
Close	100	29.9%
Positive	67	20.1%
Multifocality (339)		
Single focus	169	49.9%
Multifocal	170	50.1%
Size of the tumor		
<2 cm	149	42.6%
2–4 cm	131	37.4%
≥4 cm	70	20.0%

**Table 4:** Differences in the clinical characteristics of the patients according to the duration between the diagnosis and surgery.

Characteristics	Duration		*p* *
	<6 Months	≥6 Months	
	N (%)	N (%)	
Thyroiditis			
Positive	123 (76.4)	38 (23.6)	0.564
Negative	120 (73.6)	43 (26.4)	
Goitrous			
Positive	97 (69.8)	42 (30.2)	0.062
Negative	162 (78.6)	44 (21.4)	
ETE			
Positive	92 (79.3)	24 (20.7)	0.149
Negative	164 (73.5)	59 (26.5)	
LN			
Positive	101 (84.2)	19 (15.8)	0.004 **
Negative	161 (70.3)	68 (29.7)	
ECE			
Positive	101 (78.3)	28 (21.7)	0.589
Negative	82 (81.2)	19 (18.8)	
Lymphovascular invasion			
Positive	57 (81.5)	13 (18.6)	0.265
Negative	177 (75.0)	59 (25.0)	
Neural invasion			
Positive	2 (66.7)	1 (33.3)	0.507
Negative	78 (79.6)	20 (20.4)	
Thyroid function			
Euthyroid	218 (74.7)	74 (25.3)	
Hypothyroid	15 (68.2)	7 (31.8)	0.563
Hyperthyroid	29 (80.6)	7 (19.4)	
Multifocality			
Single focus	124 (73.4)	45 (26.6)	0.511
Multifocal	130 (76.5)	40 (23.5)	
FNA			
PTC	119 (91.5)	11 (8.5)	
Suspecouic for PTC	56 (88.9)	7 (11.1)	
Atypia of Undetermined Significance	36 (59.0)	25 (41.0)	
Benign	20 (34.5)	28 (65.5)	<0.001 **
Follicular Neoplasm	26 (92.9)	2 (7.1)	
Not diagnostic	5 (50.0)	5 (50.0)	
Tumor size			
<2 cm	108 (72.5)	41 (27.5)	0.608
2–<4 cm	99 (75.6)	32 (24.4)	
≥4 cm	55 (78.6)	15 (21.4)	
Margin			
Negative	122 (73.1)	45 (26.9)	0.589
Close	77 (77.0)	23 (23.0)	
Positive	51 (76.1)	16 (23.9)	

* Based on Chi-square test; ** statistically significant. ETC (Extrathyroidal Extension), ECE (Extracapsular Extension), LN (Lymph node), FNA (Fine Needle Aspiration), PTC (Papillary Thyroid Carcinoma).

**Table 5:** Logistic regression for the factors potentially predicting LN involvement among cases.

Parameter	B	Std. Error	95% Wald CI		Hypothesis Test			Exp (B)	95% CI for Exp (B)	
			Lower	Upper	Wald Chi-Square	df	Sig.		Lower	Upper
(Intercept)	0.398	0.0679	0.265	0.531	34.417	1	<0.001	1.489	1.304	1.701
Age category										
≥55 years	0.140	0.0719	−0.001	0.281	3.791	1	0.052	1.150	0.999	1.324
<55 years (ref)	0 a	.	.	.	.	.	.	1	.	.
BMI category										
Morbid obesity	0.066	0.0740	−0.079	0.211	0.790	1	0.374	1.068	0.924	1.235
Obesity	0.010	0.0707	−0.128	0.149	0.021	1	0.885	1.010	0.880	1.160
Overweight	0.110	0.0670	−0.022	0.241	2.676	1	0.102	1.116	0.978	1.273
Within normal (ref)	0 a	.	.	.	.	.	.	1	.	.
Tumor size										
≥4 cm	0.104	0.0646	−0.022	0.231	2.598	1	0.107	1.110	0.978	1.259
2–4 cm	0.056	0.0538	−0.050	0.161	1.074	1	0.300	1.057	0.952	1.175
<2 cm (ref)	0 a	.	.	.	.	.	.	1	.	.
FNA result										
Not diagnostic	−0.006	0.1453	−0.291	0.278	0.002	1	0.965	0.994	0.747	1.321
Benign	−0.341	0.0702	−0.478	−0.203	23.572	1	<0.001	0.711	0.620	0.816
AUS	−0.356	0.0698	−0.492	−0.219	25.966	1	<0.001	0.701	0.611	0.803
Follicular Neoplasm	−0.358	0.0924	−0.539	−0.177	14.980	1	<0.001	0.699	0.583	0.838
Suspecouic for PTC	−0.095	0.0690	−0.230	0.040	1.897	1	0.168	0.909	0.794	1.041
PTC (ref)	0 a	.	.	.	.	.	.	1	.	.
(Scale)	0.195 b	0.0148	0.168	0.226						

a (Constrained to zero because the parameter is not estimable), b (Fixed at the displayed value).

**Table 6:** Logistic regression for the factors potentially predicting ETE (Extrathyroidal Extension) among cases.

Parameter	B	Std. Error	95% Wald CI		Hypothesis Test			Exp (B)	95% CI for Exp (B)	
			Lower	Upper	Wald Chi-Square	df	Sig.		Lower	Upper
(Intercept)	0.339	0.0671	0.207	0.470	25.529	1	<0.001	1.403	1.231	1.601
Age category										
≥55 years	0.241	0.0718	0.101	0.382	11.290	1	<0.001	1.273	1.106	1.465
<55 years (ref)	0 a	.	.	.	.	.	.	1	.	.
BMI category										
Morbid obesity	0.077	0.0732	−0.066	0.221	1.107	1	0.293	1.080	0.936	1.247
Obesity	−0.056	0.0698	−0.193	0.081	0.640	1	0.424	0.946	0.825	1.084
Overweight	−0.004	0.0665	−0.134	0.127	0.003	1	0.958	0.996	0.875	1.135
Within normal (ref)	0 a	.	.	.	.	.	.	1	.	.
Tumor size										
≥4 cm	0.251	0.0644	0.125	0.378	15.221	1	<0.001	1.286	1.133	1.459
2–4 cm	0.207	0.0533	0.103	0.312	15.071	1	<0.001	1.230	1.108	1.366
<2 cm (ref)	0 a	.	.	.	.	.	.	1	.	.
FNA result										
Not diagnostic	−0.272	0.1496	−0.565	0.022	3.297	1	0.069	0.762	0.568	1.022
Benign	−0.308	0.0703	−0.446	−0.170	19.215	1	<0.001	0.735	0.640	0.843
AUS	−0.273	0.0691	−0.409	−0.138	15.670	1	<0.001	0.761	0.664	0.871
Follicular Neoplasm	−0.303	0.0922	−0.484	−0.122	10.800	1	0.001	0.739	0.617	0.885
Suspecouic for PTC	−0.148	0.0682	−0.282	−0.015	4.736	1	0.030	0.862	0.754	0.985
PTC (ref)	0 a	.	.	.	.	.	.	1	.	.
(Scale)	0.187 b	0.0144	0.161	0.217						

a (Constrained to zero because the parameter is not estimable), b (Fixed at the displayed value).

## Data Availability

The data that support the findings of this study are not publicly available due to patient confidentiality. However, the data can be made available from the corresponding author upon reasonable request.
